# Sleep disturbance is associated with perturbations in immune-inflammatory pathways in oncology outpatients undergoing chemotherapy

**DOI:** 10.1016/j.sleep.2022.11.014

**Published:** 2022-11-21

**Authors:** Alejandra Calvo-Schimmel, Kord M. Kober, Steven M. Paul, Bruce A. Cooper, Carolyn Harris, Joosun Shin, Marilyn J. Hammer, Yvette P. Conley, Vasuda Dokiparthi, Adam Olshen, Jon D. Levine, Christine Miaskowski

**Affiliations:** a Department of Physiological Nursing, University of California, San Francisco, San Francisco, CA, USA; b Dana Farber Cancer Institute, Boston, MA, USA; c School of Nursing, University of Pittsburgh, Pittsburgh, PA, USA; d Department of Epidemiology and Biostatistics, University of California, San Francisco, CA, USA; e Department of Medicine, University of California, San Francisco, San Francisco, CA, USA

**Keywords:** Cancer, Chemotherapy, Inflammation, Immune mechanisms, Insomnia, Pathway impact analysis, Sleep disturbance

## Abstract

**Objective/background:**

Sleep disturbance is a common problem in patients receiving chemotherapy. Purpose was to evaluate for perturbations in immune-inflammatory pathways between oncology patients with low versus very high levels of sleep disturbance.

**Patients/methods:**

Sleep disturbance was evaluated using the General Sleep Disturbance Scale six times over two cycles of chemotherapy. Latent profile analysis was used to identify subgroups of patients with distinct sleep disturbance profiles. Pathway impact analyses were performed in two independent samples using gene expression data obtained from RNA sequencing (n = 198) and microarray (n = 162) technologies. Fisher's combined probability test was used to identify significantly perturbed pathways between Low versus Very High sleep disturbance classes.

**Results:**

In the RNA sequencing and microarray samples, 59.1% and 51.9% of patients were in the Very High sleep disturbance class, respectively. Thirteen perturbed pathways were related to immune-inflammatory mechanisms (i.e., endocytosis, phagosome, antigen processing and presentation, natural killer cell mediated cytotoxicity, cytokine-cytokine receptor interaction, apoptosis, neutrophil extracellular trap formation, nucleotide-binding and oligomerization domain-like receptor signaling, Th17 cell differentiation, intestinal immune network for immunoglobulin A production, T-cell receptor signaling, complement and coagulation cascades, and tumor necrosis factor signaling).

**Conclusions:**

First study to identify perturbations in immune-inflammatory pathways associated with very high levels of sleep disturbance in oncology outpatients. Findings suggest that complex immune-inflammatory interactions underlie sleep disturbance.

## 1. Introduction

Sleep disturbance is an umbrella term that encompasses a variety of problems (e.g., difficulties initiating and maintaining sleep, excessive daytime somnolence). Reported by 30%–88% of patients, sleep disturbance is one of the most common symptoms associated with cancer [1–5]. Specifically, in patients undergoing chemotherapy, clinically meaningful levels of sleep disturbance are associated with higher levels of emotional problems [3–5], decreases in immune responses [4], obesity [4], cardiovascular problems [3], reductions in overall quality of life (QOL) [3,4], and higher mortality rates [3,5]. While the prevalence of and risk factors for sleep disturbance in oncology patients were described in several reviews [4,6–8], less is known about its underlying mechanism(s).

Most of the research on sleep mechanisms has examined associations between sleep problems (e.g., circadian rhythm variations, insomnia) and genomic markers (e.g., circadian clock genes) using candidate gene analyses and genome-wide association studies [9–11]. However, as noted in three reviews [9,12,13], a wide variety of underlying molecular mechanisms (e.g., immune, endocrine, ion channels) may be involved in sleep disturbance. For example, the hypothesis that an association exists between sleep disturbance and immune-inflammatory signaling pathways is supported by findings that suggest that prolonged sleep deficiency (e.g., short sleep duration) is related to stimulation of the immune system, that results in a systemic and prolonged low-grade inflammatory response [14].

One preclinical [15] and five clinical [11,16–19] studies have used pathway analytic approaches to evaluate for associations between sleep disturbance and/or insomnia and perturbations in immune-inflammatory pathways. In a preclinical study that used a chronic jet-lag paradigm to mimic long-term circadian misalignment [15], evaluation of changes in gene expression and pathway enrichment analyses were performed using liver and kidney tissues from experimental and control mice. In the liver, while circadian system-, immune disease-, and inflammation-related pathways were markedly activated, pathways associated with lipid and amino acid metabolism were repressed. Similar relationships were found in the kidney, but only for immune-related pathways. These findings suggest an association between circadian clock disorders and inflammatory mechanisms.

In the first clinical study [16], six healthy individuals were assessed prior to and after staying up all night to evaluate the effects of poor sleep on immune function in terms of cell type composition, subset specific gene expression, enriched pathways, transcriptional regulation, and cell-cell communications. The major findings suggested that poor sleep induced a pro-inflammatory state in peripheral blood; cytotoxic T-cells lost their immune activity and exhibited a phenotype associated with inflammatory disease; and cellular senescence was activated. In another study of 11 monozygotic twin pairs [19], associations between habitual short sleep duration and changes in gene expression were evaluated. Using gene set enrichment analysis, habitual short sleep was associated with downregulation of genes involved in several inflammatory pathways (e.g., interleukin (IL) signaling, leukocyte activation).

In the third study that compared four healthy controls (i.e., slept 8 h per night) to nine healthy men whose sleep was restricted to 4 h per night for five nights [18], fifteen of the 25 most up-regulated Gene Ontology pathways were related to immune function (e.g., B cell activation, IL-8 production, nuclear factor kappa B (NF-kB) signaling). Finally, in the two studies that evaluated for associations between insomnia and changes in gene expression [11,17], perturbations in signaling pathways involved in immune and inflammatory responses (e.g., NF-kB) were identified. Taken together, these findings suggest associations between various types of sleep disturbance and perturbations in several immune-inflammatory pathways. However, across these studies, sample sizes were relatively small and only healthy individuals were evaluated.

Given that sleep disturbance is a common problem in oncology patients and building on the findings in healthy individuals [11,16–19], the purpose of this study was to evaluate for perturbations in immune-inflammatory pathways across two independent samples of oncology patients who reported low versus very high levels of sleep disturbance. For this analysis, we used the results of a previously reported latent profile analysis (LPA) that identified three classes of oncology patients with distinct sleep disturbance profiles (i.e., Low (25.2%), High (50.8%), Very High (24.0%)) [1]. Using an extreme phenotype approach, perturbations in immune-inflammatory pathways between the Low and Very High sleep disturbance classes were evaluated.

## 2. Methods

### 2.1. Patients and settings

This study is part of a larger, longitudinal study of the symptom experience of oncology outpatients receiving chemotherapy [20]. Eligible patients were ≥18 years of age; had a diagnosis of breast, gastrointestinal, gynecological, or lung cancer; had received chemotherapy within the preceding four weeks; were scheduled to receive at least two additional cycles of chemotherapy; were able to read, write, and understand English; and gave written informed consent. Patients were recruited from two Comprehensive Cancer Centers, one Veteran's Affairs hospital, and four community-based oncology programs.

### 2.2. Study procedures

The study was approved by the Institutional Review Board at each of the study sites. Of the 2234 patients approached, 1343 consented to participate (60.1% response rate). The major reason for refusal was being overwhelmed with their cancer treatments. Eligible patients were approached in the infusion unit during their first or second cycle of chemotherapy by a member of the research team to discuss study participation and obtain written informed consent.

Patients completed the General Sleep Disturbance Scale (GSDS) [21] six times over two cycles of chemotherapy (i.e., prior to chemotherapy administration, approximately 1 week after chemotherapy administration, and approximately 2 weeks after chemotherapy administration). Blood for ribonucleic acid (RNA) isolation was collected at the enrollment assessment. Medical records were reviewed for disease and treatment information. For this study, a total of 717 patients provided a blood sample for the gene expression and pathway perturbation analyses (Supplemental Fig. 1). Of these 717 patients, 198 patients had their samples processed using RNA sequencing (i.e., RNA-seq sample) and 162 patients had their samples processed using microarray (i.e., microarray sample) technologies.

### 2.3. Instruments

#### 2.3.1. Demographic and clinical characteristics

Demographic information was obtained using a self-report questionnaire. Functional status was assessed using the Karnofsky Performance Status (KPS) scale [22]. The occurrence, treatment, and functional impact of 13 common medical conditions were assessed using the Self-Administered Comorbidity Questionnaire (SCQ) [23]. Alcohol consumption, behaviors, and associated problems were measured using the Alcohol Use Disorders Identification test (AUDIT) [24]. The toxicity of each patient's chemotherapy regimen was rated using the MAX2 index [25]. Medical records were reviewed for disease and treatment information.

#### 2.3.2. Sleep disturbance measure

The 21-item GSDS was designed to assess various aspects of sleep disturbance (i.e., quality, quantity, onset latency, mid and early awakenings, sleep medications, daytime sleepiness). Each item was rated from 0 (never) to 7 (everyday) numeric rating scale (NRS). GSDS total score ranges from 0 (no disturbance) to 147 (extreme sleep disturbance) [21]. A score of ≥43 indicates a clinically meaningful level of sleep disturbance. In this study, the Cronbach's alpha for the GSDS total score was 0.83.

### 2.4. Data analysis

#### 2.4.1. Latent profile analysis

In our previous study [1], LPA was used to identify subgroups of patients (i.e., latent classes) with distinct sleep disturbance profiles over the six assessments, using the GSDS scores. In brief, LPA was performed using MPlus^™^ Version 8.4 [26]. Estimation was carried out with full information maximum likelihood with standard error and a Chi square test that are robust to non-normality and non-independence of observations (“estimator = MLR”). Model fit was evaluated to identify the solution that best characterized the observed latent class structure with the Bayesian Information Criterion, Vuong-Lo-Mendell-Rubin likelihood ratio test, entropy, and latent class percentages that were large enough to be reliable [26,27]. Missing data were accommodated for with the use of the Expectation-Maximization algorithm [28]. Three latent classes were identified and named Low, High, and Very High. For the current analysis, using an extreme phenotype approach, perturbations in immune-inflammatory pathways between patients in the Low and Very High sleep disturbance classes were evaluated.

#### 2.4.2. Imputation process

Missing data for demographic and clinical characteristics were imputed by the k-nearest-neighbors method, with k = 9. For continuous variables the Euclidean distance was used to find the nearest neighbors. The imputed value was the weighted average of the nearest neighbors, with each weight originally exp(-dist(x,j)), after which the weights were scaled to one. For categorical variables, distance was 0 if the predictor and the neighbor had the same value and 1 if they did not. The imputed value was the mode of the nearest neighbors.

#### 2.4.3. Demographic and clinical data

Demographic and clinical data from the two patient samples (i.e., RNA-seq, microarray) were analyzed separately. Differences in demographic and clinical characteristics between the Low and Very High sleep disturbance classes were evaluated using parametric and non-parametric tests. Significance corresponded to a p-value of < .05. Characteristics included in the final model were selected using a backwards stepwise logistic regression approach based on the likelihood ratio test (LRT). The area under the curve (AUC) of the receiver operating characteristic (ROC) curves was used to gauge the overall adequacy of the logistic regression model for each sample [29]. All these analyses were performed using R (version 4.1) [30].

#### 2.4.4. Differential expression and pathway impact analyses (PIA)

Differential expression was quantified using generalized linear models that were implemented separately for each sample (i.e., using edgeR [31] for the RNA-seq sample and limma [32] for the microarray sample). These analyses were adjusted for demographic and clinical characteristics that were significantly different between the Low versus the Very High sleep disturbance classes. In addition, the models included surrogate variables not associated with class memberships to adjust for variations due to unmeasured sources [33,34]. Expression loci were annotated with Entrez gene identifiers. Gene symbols were derived from the HUGO Gene Nomenclature Committee resource database [35]. The differential expression results were summarized as the log fold-change and p-value for each gene. Only genes that had a common direction of expression (i.e., the same sign for the log fold-change) across the two samples were retained for subsequent analyses. Common genes were matched using gene symbol.

To interpret the results in the context of immune-inflammatory-related mechanisms, we used PIA to identify perturbed pathways associated with differences in gene expression between the Low and Very High sleep disturbance classes. The PIA included potentially important biological factors (e.g., gene-gene interactions, flow signals in a pathway, pathway topologies), as well as the magnitude (i.e., log fold-change) and *p*-values from the differential expression analysis for each sample [36]. The PIA included the results of the differential expression analyses for all of the genes (i.e., cutoff free) that had a common direction of differential expression to determine probability of pathway perturbations (pPERT) using Pathway Express [37]. A total of 224 signaling pathways were defined using the Kyoto Encyclopedia of Genes and Genomes (KEGG) database [38]. For each sample, a separate test was performed for each pathway. Then, Fisher's Combined Probability method was used to combine these test results to obtain a single test (global) of the null hypothesis [39,40]. The significance of the combined transcriptome-wide PIA analysis was assessed using a false discovery rate (FDR) of 0.015 [41]. Finally, we evaluated these results for perturbed immune-inflammatory signaling pathways.

## 3. Results

### 3.1. RNA-seq performance

Of the 198 patients in the RNA-seq sample, 81 were in the Low and 117 were in the Very High classes (Table 1). Median library threshold size was 6.21. Following the application of quality control filters, 10,825 genes were included in the final analysis. The common dispersion was estimated as 0.21, yielding a biological coefficient of variation of 0.45 well within the expected value for clinical samples [42].

### 3.2. Microarray performance

Of the 162 patients in the microarray sample, 78 patients were in the Low and 84 were in the Very High sleep disturbance classes (Table 2). All of these samples demonstrated good hybridization performance for biotin, background negative, and positive control assays on the arrays. Limma was used for background correction, quantile normalization, and log2 transformation [43]. Following quality control filters, 42,921 loci were included in the final analysis.

### 3.3. Differences in demographic and clinical characteristics

Of the 198 patients with phenotypic data in the RNA-seq sample (Table 1), compared to the Low class, the Very High class was significantly younger and more likely to be female, live alone, and had a lower annual income. Patients in the Very High class had lower KPS scores; a higher number of comorbidities; higher SCQ scores; higher MAX2 scores; were more likely to self-report a diagnosis of anemia or blood disease, depression, or back pain; and were less likely to have gastrointestinal cancer and more likely to have gynecological cancer.

Of the 162 patients with phenotypic data in the microarray sample (Table 2), compared to the Low class, the Very High class was significantly younger; more likely to be female and have child care responsibilities; and less likely to be married/partnered, employed, and exercise on a regular basis. Patients in the Very High class had lower KPS scores; a higher body mass index (BMI); a higher number of comorbidities; higher SCQ scores; higher MAX2 scores; were more likely to self-report a diagnosis of anemia or blood disease or depression; and were more likely to receive an antiemetic regimen containing a neurokin-1 receptor antagonist in combination with other two antiemetics.

### 3.4. Logistic regression analyses

For the RNA-seq sample (Table 3), six variables were retained in the final logistic regression model (i.e., age, gender, lives alone, income, KPS score, SCQ score) and were used as covariates in the gene expression analysis. Patients who were younger, female, lived alone, had a lower KPS score, and a higher SCQ score were more likely to belong to the Very High class. Compared to patients who had an income of $30,000 or less, patients with an annual income between $30,000 and $70,000 and between $70,000 and $100,000 had a 77% and 73% decrease in the odds of belonging in the Very High class respectively.

For the microarray sample (Table 3), eight variables were retained in the final logistic regression model (i.e., age, married or partnered, currently employed, exercise on a regular basis, BMI, MAX2 score, KPS score, self-reported diagnosis of depression) and were used as covariates in the gene expression analysis. Patients who were not married or partnered and had a lower KPS score were more likely to belong to the Very High class. Patients who exercised on a regular basis had a 75% decrease in the odds of belonging to the Very High class. Having a self-reported diagnosis of depression was associated with a 5.19 times increase in the odds of belonging in the Very High class.

### 3.5. Differentially expressed genes and pathways between the Low and Very High classes

Of the 14 surrogate variables identified for the RNA-seq sample, one was associated with sleep disturbance scores and was excluded from the final model. The final differential expression model for this sample included 13 surrogate variables and six significant demographic and clinical characteristics.

Of the 15 surrogate variables identified for the microarray sample, one was associated with sleep disturbance scores and was excluded from the final model. The final differential expression model for this sample included 14 surrogate variables and eight demographic and clinical characteristics. For both samples, a total of 4090 genes were included in the PIA analyses. As shown in Table 4, using Fisher's combined probability method, across the two samples, thirteen KEGG signaling pathways related to immune-inflammatory mechanisms were significantly perturbed.

## 4. Discussion

This study is the first to describe perturbations in immune-inflammatory pathways associated with sleep disturbance in oncology outpatients receiving chemotherapy. Our findings extend previous preclinical [15] and clinical [11,16–19] research and suggests that the sleep disturbance reported by patients receiving chemotherapy is associated with changes in a number of immune-inflammatory pathways. Our previous paper described in detail the phenotypic characteristics associated with membership in the Very High sleep disturbance class [1]. The remainder of this discussion focuses on the perturbed KEGG pathways that were identified, namely: endocytosis, phagosome, antigen processing and presentation, natural killer (NK) cell mediated cytotoxicity, cytokine-cytokine receptor interaction, apoptosis, neutrophil extracellular trap formation, nucleotide-binding and oligomerization domain-like (NOD-like) receptor signaling, Th17 cell differentiation, intestinal immune network for immunoglobulin (Ig) A production, T-cell receptor (TCR) signaling, complement and coagulation cascades, and tumor necrosis factor (TNF) signaling and their association with sleep disturbance.

### 4.1. Endocytosis

Endocytosis is a process through which various substances are internalized into a cell. As noted in one review [44], recent evidence suggests that endocytosis occurs across the blood brain barrier (BBB) during sleep. This process results in the clearance of waste products in the perivascular space. Endocytosis may be of clinical importance. For example, in a preclinical study that evaluated if cerebrospinal fluid clearance was increased during sleep [45], findings suggest that the enhanced removal of neurotoxic waste products that accumulate in the awake central nervous system may be a key component of restorative sleep.

Equally important, loss of sleep increases the activity of several pro-inflammatory cytokines (e.g., TNF, IL6) that can lead to increased permeability of the BBB [44,46]. In addition, while the sleep-wake cycle effects endocytotic processes, levels of endocytosis appear to regulate sleep. For example, in one study of Drosophila [46], blocking endocytosis resulted in an increase in total sleep time.

### 4.2. Phagosome

Phagocytosis is a process through which cells recognize, engulf, and digest a variety of large particles (e.g., apoptotic cells, cellular debris). Within the central nervous system, phagocytosis plays a critical role in the proper development of neural circuits and the maintenance of homeostasis [47]. Specifically, microglial cells are the immune cells that are involved in the phagocytosis of cellular debris and the “orchestration” of neuroinflammation within the brain [48]. Recent hypotheses suggest that inflammation and neurodegenerative processes may occur if phagocytotic processes fail [47].

Albeit limited, a growing body of evidence suggests that microglia play a role in a variety of sleep disorders [48]. For example, during an acute infection with its associated “sickness behavior”, sleep disturbance and fatigue are common symptoms. These symptoms may be associated with an increase in the production of cytokines by microglia [49]. In addition, alterations in the homeostatic functioning of microglia may be one of the underlying mechanisms for narcolepsy [48]. Increases in inflammation and alterations in microglia's phagocytic activity may result in the degeneration of hypocretin neurons that are implicated in the pathophysiology of narcolepsy.

Of note, total or partial sleep disturbance can alter phagocytic activity and increase inflammatory processes [50]. For example, in a preclinical study that investigated whether microglia phagocytose synapses around the time of sleep onset or during the light phase to help facilitate sleep [51], findings suggest that through a process of phagocytosis, weak synapses are eliminated during every phase of sleep.

### 4.3. Antigen processing and presentation

Antigen processing and presentation is a molecular mechanism by which whole antigens are broken down and loaded onto major histocompatibility complex (MHC) molecules for display on the surface of T and B cells [52–54]. While MHC class I molecules “report” on endogenous events (e.g., intracellular bacteria, cellular transformation) to CD8^+^ T cells, MHC class II molecules present exogenous antigens to CD4^+^ T cells [53]. As noted in a number of reviews [55–57], sleep deprivation is associated with alterations in innate and adaptive immune responses. Specifically, sleep deprivation contributes to altered antigen presentation; lowered Th1 responses; higher Th2 responses; and decreases in antibody production. These changes lead to chronic inflammation and increased risk for and/or worsening of several chronic conditions (e.g., cancer, Alzheimer's disease, cardiovascular disease, diabetes).

### 4.4. NK cell mediated cytotoxicity

NK cells are a family of cytotoxic lymphocytes that lyse cancer and virally infected cells. Once activated, NK cells can produce biochemical signals and cytokines to stimulate adaptive immune responses [58]. As noted in preclinical [59] and clinical [60,61] studies, sleep disturbance was associated with a significant decrease in NK cell counts. This reduction was associated with increases in levels of TNF-alpha, that suppressed the expression of circadian rhythm genes (i.e., PER1, PER2, PER3) in NK cells.

### 4.5. Cytokine-cytokine receptor interaction

Cytokines are a diverse group of soluble extracellular proteins that are involved in the regulation of inflammatory processes [62,63]. Cytokines are released in response to a stimulus (e.g., chemotherapy) and use multiple signaling pathways [64]. Upon release, they bind to specific surface receptors; activate signal transduction pathways; and exert a response on target cells [63,65]. Cytokine-cytokine receptor interactions are complex processes that allow cytokines to produce a variety of biological responses with a limited set of surface receptors and effector signaling molecules [65].

Across numerous reviews [56,66], evidence exists to support a role for cytokines in physiologic regulation of sleep, as well as in pathophysiologic processes. In terms of cancer, as noted in one review [67], both patients and survivors experience a variety of sleep problems including insomnia, hypersomnia, somnolence syndrome, and nightmares. While multiple mechanisms are likely to contribute to these heterogenous sleep problems, cytokines released from the tumor and associated treatments can enter the central nervous system, bind to cytokine transporters at the BBB and be transported to the brain. Once in the brain, these cytokines can activate microglia and lead to alterations in sleep and behavior.

### 4.6. Apoptosis

Apoptosis or programmed cell death, is a crucial homeostatic mechanism that is involved in normal cell turnover, as well as in the death of cells that are damaged by external agents (e.g., chemotherapy) [68]. In terms of sleep disturbance, one area of research is focused on the effects of intermittent hypoxia associated with obstructive sleep apnea. For example, in a murine model of sleep apnea [69], irreversible and functionally significant injury was found in two wake active neural groups (i.e., dopaminergic neurons in the periaqueductal gray; noradrenergic neurons in the locus coeruleus. Equally intriguing is the emerging evidence on the actions of melatonin, a neurohormone that regulates circadian rhythm and sleep-wake cycles. Depending on physiologic conditions, melatonin can promote cell death or survival [70].

### 4.7. Neutrophil extracellular trap formation

Neutrophil extracellular traps (NETs) are extracellular protein-binding traps composed of DNA, proteins, and enzymes. The formation of NETs, a process called NETosis, is a specific form of cell death performed by neutrophils. While NETs play a crucial role in our innate immune response, recent evidence suggests that they are involved in a variety of inflammatory diseases (e.g., diabetes, rheumatoid arthritis) [71]. While direct evidence to support associations between sleep disturbance and NETs was not found, insomnia disorders and obstructive sleep apnea are associated with increases in inflammatory responses [66]. In addition, increases in inflammatory responses were found in oncology patients with sleep disturbance [72].

### 4.8. NOD-like receptor signaling pathway

NOD-like receptors are a group of intracellular pattern-recognition proteins that play a critical role in the regulation of immune and inflammatory responses through a variety of mechanisms (e.g., formation of inflammasomes, activation of NF-κ B and mitogen-activated protein kinases (MAPK) pathways, cytokine production, apoptosis) [73]. When NOD-like receptors detect “non-self” molecules produced by pathogens or endogenous damage-associated molecular patterns, they initiate an inflammatory response that facilitates tissue repair/regeneration and homeostasis [74].

Of note, sustained activation of one of the NOD-like receptors, namely, leucine-rich repeat and pyrin domain containing protein 3 (i.e., NLRP3 inflammasome), with exogenous or endogenous triggers, exacerbates a number of chronic inflammatory diseases (e.g., obesity, type 2 diabetes mellitus, atherosclerosis) [74]. In terms of sleep disturbance, NLRP3 regulation within cells exhibits a circadian rhythm. Preclinical studies have described associations between alterations in NLRP3 signaling and sleep regulation [75], chronic obstructive sleep apnea [76], and sleep deprivation [77]. In a study of patients with insomnia with objective short sleep duration [78], increases in plasma levels of NLRP3 were associated with less total sleep time and longer wake after sleep onset times.

### 4.9. Th17 cell differentiation

Interleukin-17 producing CD4^+^ helper T cells (i.e., Th17 cells) are pro-inflammatory immune cells that protect against bacterial and fungal infections [79]. Alterations in Th17 cells are associated with a number of inflammatory diseases [80–82]. In terms of sleep disturbance, evidence suggests that lineage specification of Th17 cells is under direct circadian control [83]. In addition, in an experimental model of multiple sclerosis [84], melatonin, a hormone that is involved in the regulation of circadian rhythm, interfered with the differentiation of Th17 cells.

### 4.10. Intestinal immune network for IgA production

The intestine is the largest lymphoid tissue in the body. One striking feature of intestinal immunity is its ability to generate large amounts of noninflammatory IgA antibodies that serve as the first line of defense against bacteria and other pathogens. This immunologic barrier is achieved through IgA antibodies that prevent pathogens from adhering to intestinal epithelial cells; suppress the growth and virulence of pathogens; and neutralize toxins [85]. As part of humoral immunity, a primary function of IgA is to maintain the functioning of a healthy intestinal microbiota.

Recent evidence suggests that a bidirectional relationship exists between the immune system in the gastrointestinal tract and the brain. For example, while gut microbiota may modulate brain function, the brain may alter the gut's microbiota by changing intestinal permeability and gastrointestinal motility [86]. Recent reviews have noted that the gut microbiome is essential for the maintenance of normal sleep. In addition, abnormal sleep patterns and alterations in the duration of sleep can effect the composition, diversity, and function of the gut microbiota [87,88]. Finally, interventions that target the gut microbiome may improve insomnia [89].

### 4.11. TCR signaling pathway

The TCR is an antigen recognition signaling pathway that is composed of membrane proteins, co-receptors, kinases, and ligands. Following interaction with a foreign antigen that is associated with MHC molecules, a cascade of signaling events occur that results in T cell proliferation, cytokine production, and differentiation of T cells into effector cells [90]. Numerous immunologic functions, including the type and magnitude of various immune responses, are dependent on circadian rhythms and sleep [12]. In a study that investigated how sleep and circadian rhythm influence the number and function of natural regulatory T cells in healthy adults [91], these T cells displayed a circadian rhythm with the highest cell counts during the night and the lowest levels during the day. In addition, sleep deprivation decreased T cell proliferation.

### 4.12. Complement and coagulation cascades

The complement system is a proteolytic cascade that mediates humoral immunity [92]. The coagulation system is an amplification cascade that maintains hemostasis [93]. Given that these two systems share a common ancestry, evidence suggests that cross talk occurs between these two systems; particularly in the setting of inflammation [93]. In terms of sleep disturbance, as noted in one review [94], the complement system exhibits circadian properties. While not completely understood, disruption in circadian rhythms can induce dysregulation of pro-inflammatory cytokines and complement factors. Of note, in a recent study [95], compared to healthy controls, patients with obstructive sleep apnea had elevated morning and evening levels of plasma C3a. In addition, elevated levels of C3a were correlated with increased severity of obstructive sleep apnea.

### 4.13. TNF signaling pathway

TNF is a soluble pro-inflammatory cytokine member of the TNF ligand family [96]. TNF can modulate multiple signaling pathways (e.g., apoptosis, inflammation) [97]. As noted in one review [98], in healthy animals and albeit limited studies in healthy humans, acute enhancement or inhibition of TNF in the brain, promotes and inhibits sleep. In addition, most of the clinical studies on the role of TNF and sleep, evaluated patients with chronic elevations in systemic TNF. Across these studies, these patients had disruptions in sleep and normalization of their TNF levels resulted in improvements sleep [98]. Equally important, TNF-alpha plays an important role in the regulation of circadian rhythms by regulating the transcription of clock genes [99].

### 4.14. Strengths and limitations

This study had several strengths including: a relatively large sample size; rigorous quality controls; and results from independent tests across two samples. However, some limitations must be acknowledged. Because of its cross-sectional design, longitudinal studies are needed that assess for associations between changes in sleep disturbance before, during, and after chemotherapy and pathway perturbations. Second, given that this study is the first to evaluate for associations between sleep disturbance and pathway perturbations in oncology patients undergoing chemotherapy, our findings warrant confirmation in independent samples. Third, this study evaluated for sleep disturbance using a single subjective measure. Future research should explore associations between subjectively and objectively measured sleep disturbance and pathway perturbations. Fourth, we did not evaluate for the use of sleep medications. Lastly, because sleep disturbance was assessed only during chemotherapy, future studies should attempt to replicate these findings in oncology patients undergoing other types of treatment (e.g., radiation therapy, immunotherapy, surgery).

### 4.15. Conclusions

Consistent with the findings summarized above for preclinical and clinical research in patients without cancer, our findings suggest strong associations between sleep disturbance and perturbations in several immune-inflammatory pathways in oncology patients receiving chemotherapy. However, as illustrated in this analysis, associations between sleep disturbance and immune-inflammatory mechanisms are complex. Therefore, additional research is needed to elucidate the role of these mechanisms in the development and maintenance of sleep disturbance in patients with cancer.

## Supplementary Material

Supp File 1

## Figures and Tables

**Table 1:** Differences in demographic and clinical characteristics between patients in RNA-Seq sample with low and very high levels of sleep disturbance.

Characteristic	Low (0)	Very High (1)	Statistics
	40.9% (n = 81)	59.1% (n = 117)	
	Mean (SD)	Mean (SD)	
Age (years)	60.1 (10.8)	55.0 (12.4)	t = 2.99, p = .003
Education (years)	16.1 (2.9)	15.8 (3.1)	t = 0.70, p = .485
Body mass index (kg/m^2^)	25.8 (4.4)	26.8 (6.0)	t = −1.32, p = .188
Karnofsky Performance Status score	82.6 (12.8)	73.4 (12.2)	t = 5.08, p < .001
Number of comorbidities	2.2 (1.2)	3.1 (1.7)	t = −4.25, p < .001
Self-administered Comorbidity Questionnaire score	4.9 (2.8)	7.4 (4.0)	t = −4.83, p < .001
Alcohol Use Disorders Identification Test score	3.0 (2.5)	2.7 (2.1)	t = 0.70, p = .483
Time since diagnosis (years)	1.7 (2.8)	1.5 (3.0)	U, p = .683
Time since diagnosis (median)	0.44	0.53	
Number of prior cancer treatments	1.4 (1.4)	1.5 (1.4)	t = −0.64, p = .521
Number of metastatic sites including lymph node involvement	1.3 (1.2)	1.2 (1.1)	t = 1.06, p = .293
Number of metastatic sites excluding lymph node involvement	0.8 (1.0)	0.7 (1.0)	t = 1.09, p = .275
MAX2 score	0.16 (0.08)	0.19 (0.08)	t = −2.23, p = .027
	% (n)	% (n)	
Gender			
Female	59.3 (48)	82.9 (97)	FE, p < .001
Male	40.7 (33)	17.1 (13)	
Ethnicity			
White	64.2 (52)	61.5 (72)	Х^2^ = 2.27, p = .519
Black	16.0 (13)	13.7 (16)	
Asian or Pacific Islander	9.9 (8)	7.7 (9)	
Hispanic mixed or other	9.9 (8)	17.1 (20)	
Married or partnered (% yes)	64.2 (52)	57.3 (67)	FE, p = .377
Lives alone (% yes)	16.0 (13)	34.2 (40)	FE, p = .005
Childcare responsibilities (% yes)	16.0 (13)	27.4 (32)	FE, p = .084
Care of adult responsibilities (% yes)	2.5 (2)	6.0 (7)	FE, p = .314
Currently employed (% yes)	38.3 (31)	24.8 (29)	FE, p = .059
Income			
<$30,000	11.1 (9)	35.0 (41)	
$30,000 to <$70,000	22.2 (18)	16.2 (19)	
$70,000 to <$100,000	28.4 (23)	15.4 (18)	
≥$100,000	38.3 (31)	33.3 (39)	U, p = .011
Specific comorbidities (% yes)			
Heart disease	4.9 (4)	9.4 (11)	FE, p = .286
High blood pressure	34.6 (28)	39.3 (46)	FE, p = .551
Lung disease	8.6 (7)	12.8 (15)	FE, p = .491
Diabetes	11.1 (9)	13.7 (16)	FE, p = .667
Ulcer or stomach disease	1.2 (1)	5.1 (6)	FE, p = .244
Kidney disease	0.0 (0)	2.6 (3)	n/a
Liver disease	7.4 (6)	5.1 (6)	FE, p = .554
Anemia or blood disease	4.9 (4)	14.5 (17)	FE, p = .035
Depression	11.1 (9)	35.0 (41)	FE, p < .001
Osteoarthritis	9.9 (8)	18.8 (22)	FE, p = .107
Back pain	22.2 (18)	51.3 (60)	FE, p < .001
Rheumatoid arthritis	2.5 (2)	5.1 (6)	FE, p = .475
Exercise on a regular basis (% yes)	70.4 (57)	61.5 (72)	FE, p = .227
Smoking current or history of (% yes)	40.7 (33)	37.6 (44)	FE, p = .660
Cancer diagnosis			Х^2^ = 11.68, p = .009
Breast	33.3 (27)	41.9 (49)	NS
Gastrointestinal	46.9 (38)	25.6 (30)	0 > 1
Gynecological	7.4 (6)	18.8 (22)	0 < 1
Lung	12.3 (10)	13.7 (16)	NS
Type of prior cancer treatment			
No prior treatment	37.0 (30)	23.1 (27)	Х^2^ = 7.28, p = .063
Only surgery, CTX, or RT	32.1 (26)	46.2 (54)	
Surgery & CTX, or surgery & RT, or CTX & RT	22.2 (18)	17.1 (20)	
Surgery & CTX & RT	8.6 (7)	13.7 (16)	
CTX cycle length			
14-day cycle	51.9 (42)	42.7 (50)	U, p = .135
21-day cycle	43.2 (35)	47.0 (55)	
28-day cycle	4.9 (4)	10.3 (12)	
Emetogenicity of CTX			
Minimal/low	12.3 (10)	15.4 (18)	U, p = .917
Moderate	69.1 (56)	64.1 (75)	
High	18.5 (15)	20.5 (24)	
Antiemetic regimens			
None	6.2 (5)	4.3 (5)	Х^2^ = 6.58, p = .087
Steroid alone or serotonin receptor antagonist alone	7.4 (6)	20.5 (24)	
Serotonin receptor antagonist and steroid	55.6 (45)	47.0 (55)	
NK-1 receptor antagonist and two other antiemetics	30.9 (25)	28.2 (33)	

Abbreviations: CTX = chemotherapy; FE = Fisher's exact test; kg = kilograms; m^2^ = meter squared, n/a = not applicable; NK-1 = neurokinin-1; NS = not significant; RNA = ribonucleic acid, RT = radiation therapy; U = Mann-Whitney *U* test.

**Table 2:** Differences in demographic and clinical characteristics between patients in the microarray sample with low and very high levels of sleep disturbance.

Characteristic	Low (0)	Very High (1)	Statistics
	48.1% (n = 78)	51.9% (n = 84)	
	Mean (SD)	Mean (SD)	
Age (years)	59.8 (10.8)	55.1 (11.6)	t = 2.69, p = .008
Education (years)	16.8 (3.1)	16.4 (2.9)	t = 0.93, p = .352
Body mass index (kg/m^2^)	25.4 (5.4)	28.6 (6.8)	t = −3.29, p = .001
Karnofsky Performance Status score	87.4 (8.4)	76.1 (11.2)	t = 7.24, p < .001
Number of comorbidities	2.1 (1.2)	2.9 (1.5)	t = −3.49, p < .001
Self-administered Comorbidity Questionnaire score	4.5 (2.3)	6.7 (3.2)	t = −5.10, p < .001
Alcohol Use Disorders Identification Test score	2.9 (1.5)	3.2 (2.7)	t = −0.87, p = .383
Time since diagnosis (years)	2.1 (3.5)	2.5 (3.8)	U, p = .330
Time since diagnosis (median)	0.44	0.45	
Number of prior cancer treatments	1.8 (1.8)	2.0 (1.6)	t = −0.54, p = .590
Number of metastatic sites including lymph node involvement	1.4 (1.3)	1.1 (1.3)	t = 1.31, p = .193
Number of metastatic sites excluding lymph node involvement	0.9 (1.1)	0.8 (1.1)	t = 0.91, p = .366
MAX2 score	0.14 (0.08)	0.17 (0.08)	t = −2.10, p = .037
	% (n)	% (n)	
Gender			
Female	70.5 (55)	84.5 (71)	FE, p = .038
Male	29.5 (23)	15.5 (13)	
Ethnicity			
White	75.6 (59)	70.2 (59)	Х^2^ = 1.90, p = .594
Black	12.8 (10)	10.7 (9)	
Asian or Pacific Islander	5.1 (4)	7.1 (6)	
Hispanic mixed or other	6.4 (5)	11.9 (10)	
Married or partnered (% yes)	75.6 (59)	56.0 (47)	FE, p = .013
Lives alone (% yes)	19.2 (15)	22.6 (19)	FE, p = .700
Childcare responsibilities (% yes)	16.7 (13)	31.0 (26)	FE, p = .043
Care of adult responsibilities (% yes)	6.4 (5)	8.3 (7)	FE, p = .768
Currently employed (% yes)	46.2 (36)	25.0 (21)	FE, p = .005
Income			
<$30,000	17.9 (14)	28.6 (24)	U, p = .082
$30,000 to <$70,000	16.7 (13)	19.0 (16)	
$70,000 to <$100,000	17.9 (14)	15.5 (13)	
≥$100,000	47.4 (37)	36.9 (31)	
Specific comorbidities (% yes)			
Heart disease	6.4 (5)	10.7 (9)	FE, p = .407
High blood pressure	28.2 (22)	33.3 (28)	FE, p = .501
Lung disease	11.5 (9)	9.5 (8)	FE, p = .799
Diabetes	7.7 (6)	9.5 (8)	FE, p = .783
Ulcer or stomach disease	0.0 (0)	6.0 (5)	n/a
Kidney disease	1.3 (1)	1.2 (1)	FE, p = 1.000
Liver disease	5.1 (4)	1.2 (1)	FE, p = .197
Anemia or blood disease	9.0 (7)	22.6 (19)	FE, p = .020
Depression	7.7 (6)	41.7 (35)	FE, p < .001
Osteoarthritis	10.3 (8)	19.0 (16)	FE, p = .127
Back pain	20.5 (16)	28.6 (24)	FE, p = .276
Rheumatoid arthritis	6.4 (5)	6.0 (5)	FE, p = 1.000
Exercise on a regular basis (% yes)	78.2 (61)	56.0 (47)	FE, p = .003
Smoking current or history of (% yes)	34.6 (27)	36.9 (31)	FE, p = .870
Cancer diagnosis			
Breast	32.1 (25)	47.6 (40)	Х^2^ = 6.55, p = .088
Gastrointestinal	33.3 (26)	20.2 (17)	
Gynecological	21.8 (17)	25.0 (21)	
Lung	12.8 (10)	7.1 (6)	
Type of prior cancer treatment			
No prior treatment	26.9 (21)	15.5 (13)	Х^2^ = 6.04, p = .110
Only surgery, CTX, or RT	34.6 (27)	42.9 (36)	
Surgery & CTX, or surgery & RT, or CTX & RT	24.4 (19)	17.9 (15)	
Surgery & CTX & RT	14.1 (11)	23.8 (20)	
CTX cycle length			
14-day cycle	37.2 (29)	39.3 (33)	U, p = .705
21-day cycle	55.1 (43)	54.8 (46)	
28-day cycle	7.7 (6)	6.0 (5)	
Emetogenicity of CTX			
Minimal/low	33.3 (26)	23.8 (20)	U, p = .098
Moderate	55.1 (43)	57.1 (48)	
High	11.5 (9)	19.0 (16)	
Antiemetic regimens			Х^2^ = 11.38, p = .010
None	16.7 (13)	10.7 (9)	NS
Steroid alone or serotonin receptor antagonist alone	25.6 (20)	22.6 (19)	NS
Serotonin receptor antagonist and steroid	48.7 (38)	36.9 (31)	NS
NK-1 receptor antagonist and two other antiemetics	9.0 (7)	29.8 (25)	0 < 1

Abbreviations: CTX = chemotherapy; FE = Fisher's exact test; kg = kilograms; m^2^ = meter squared, n/a = not applicable; NK-1 = neurokinin-1; NS = not significant; RT = radiation therapy; U = Mann-Whitney *U* test.

**Table 3:** Multiple logistic regression analyses predicting very high sleep disturbance class membership.

RNA-seq Sample (n = 198)			

Predictors	Odds Ratio	95% CI	p-value
Age	0.94	0.91, 0.97	<0.001
Gender (female)	3.22	1.46, 7.37	0.004
Lives alone (% yes)	2.48	1.03, 6.30	0.048
Income			
<$30,000	1.00		
$30,000 to <$70,000	0.23	0.07, 0.73	0.015
$70,000 to <$100,000	0.27	0.08, 0.89	0.035
≥$100,000	0.46	0.15, 1.38	0.173
Karnofsky Performance Status score	0.97	0.94, 0.99	0.015
Self-administered Comorbidity Questionnaire score	1.68	1.28, 2.26	<0.001
Overall model fit: AUC of the ROC = 0.825			
Microarray Sample (n = 162)			
Predictors	Odds Ratio	95% CI	p-value

Age	0.96	0.92, 1.00	0.073
Married or partnered (% yes)	0.27	0.10, 0.66	0.005
Currently employed (% yes)	0.44	0.17, 1.12	0.091
Exercise on a regular basis (% yes)	0.25	0.09, 0.63	0.004
Body mass index	1.07	1.00, 1.16	0.066
MAX2 score	70.45	0.41, 16478.99	0.113
Karnofsky Performance Status score	0.91	0.87, 0.95	<0.001
Depression (% yes)	5.19	1.67, 18.50	0.007
Overall model fit: AUC of the ROC = 0.883			

Abbreviations: AUC = area under the curve; CI = confidence interval; ROC = receiver operating characteristic.

**Table 4:** Perturbed immune-inflammatory KEGG pathways between patients in the low and very high sleep disturbance classes.

Pathway ID	Pathway Name	Combined Analysis Statistics
hsa04144	Endocytosis	X^2^ = 30.41, FDR <0.001
hsa04145	Phagosome	X^2^ = 30.41, FDR <0.001
hsa04612	Antigen processing and presentation	X^2^ = 30.41, FDR <0.001
hsa04650	Natural killer cell mediated cytotoxicity	X^2^ = 25.13, FDR <0.001
hsa04060	Cytokine-cytokine receptor interaction	X^2^ = 23.74, FDR = 0.001
hsa04210	Apoptosis	X^2^ = 23.41, FDR = 0.001
hsa04613	Neutrophil extracellular trap formation	X^2^ = 19.81, FDR = 0.003
hsa04621	NOD-like receptor signaling pathway	X^2^ = 19.08, FDR = 0.003
hsa04659	Th17 cell differentiation	X^2^ = 19.02, FDR = 0.003
hsa04672	Intestinal immune network for IgA production	X^2^ = 18.06, FDR = 0.004
hsa04660	T-cell receptor signaling pathway	X^2^ = 17.57, FDR = 0.005
hsa04610	Complement and coagulation cascades	X^2^ = 16.76, FDR = 0.006
hsa04668	TNF signaling pathway	X^2^ = 15.41, FDR = 0.010

Abbreviations: FDR = false discovery rate; ID = identification; IgA = immunoglobulin A; KEGG = Kyoto Encyclopedia of Genes and Genomes; NOD = nucleotide-binding oligomerization domain-like; Th17 cell = T cell 17 helper; TNF = tumor necrosis factor.
