# Supplementary material for: Sleep disturbance is associated with perturbations in immune-inflammatory pathways in oncology outpatients undergoing chemotherapy
Source: Sleep Med. Author manuscript; Available in PMC 2024 Jun 26. (PMC11200329; doi:10.1016/j.sleep.2022.11.014)
Supplement: Supp File 1 [file NIHMS2000470-supplement-Supp_File_1.docx]

Supplementary Figure 1: Flow diagram of the number of patients available for phenotypic and gene

expression (GE) analyses of sleep disturbance (SD) classes.
